# Depression among patients with chronic kidney disease, associated factors, and predictors: a cross-sectional study

**DOI:** 10.1186/s12888-023-05249-y

**Published:** 2023-10-10

**Authors:** Mandreker Bahall, George Legall, Carlyle Lalla

**Affiliations:** 1 University of the West Indies, Eric Williams Medical Sciences Complex, Mt. Hope, Trinidad and Tobago; 2 San Fernando General Hospital, Chancery Lane, San Fernando, Trinidad and Tobago

**Keywords:** Depression, PHQ-9, Chronic kidney disease, Etiologies, Predictors

## Abstract

**Background:**

Depression with diverse etiologies is exacerbated by chronic diseases, such as chronic kidney disease (CKD), coronary artery disease (CAD), cancer, diabetes mellitus, and hypertension. This study aimed to analyse depression, its associations, and predictors among patients attending the kidney clinic of a teaching hospital.

**Methods:**

Data were collected from 01 August 2017 to 30 September 2017 via face-to-face interviews and examination of the medical records of a convenience sample of 314 patients. The patients were categorised broadly as stages I and II with an estimated glomerular filtration rate (eGFR) > 60 mls/min/1.73 m^2^, and with stages III, IV, and V or GFR ≤ 60 mls/ min/1.73 m^2^ (or CKD). The Patient Health Questionnaire (PHQ)-9 was the data collection instrument for depression-related data.

**Results:**

Participants were predominantly male (n = 179; 57.0%), aged over 60 years (n = 211; 67.2%), Indo-Trinbagonian (n = 237; 75.5%), and with stages III, IV, and V CKD. The two leading comorbid conditions were hypertension (83.4%) and diabetes mellitus (56.1%). Of the 261 (83.1%) patients with recorded eGFR, 113 (43.3%) had Stage III CKD. The mean depression (PHQ-9) score was 13.0/27 ($\pm$9.15), with 306 (97.5%) patients diagnosed as having depression with the following severities: *mild * (n = 116; 37.9%), *moderate* (n = 138, 45.1%), *moderately severe* (n = 38; 12.4%), and *severe* (n = 14; 4.6%). Depression was independent of sex. Nine sociodemographic variables were associated with depression; however, ‘*level of education’, was the only predictor of depression with greater severity associated with lower levels of education.* eGFR was negatively correlated with the PHQ-9 scores (Pearson’s correlation, ***r*** = -0.144, p = 0.022). At least 78.3% of the patients who self-reported no depression had clinical depression (moderate, moderately severe, or severe) PHQ-9 scores ≥ 10.

**Conclusion:**

Depression was a significant comorbidity among patients with CKD, with the majority displaying clinical depression. “*Level of education”* was the only predictor of depression. Self-reported depression is an unreliable method for evaluating clinical depression.

## Background

Chronic kidney disease (CKD) is irreversible damage to the kidney lasting > 3 months. Worldwide, CKD is quite prevalent, impacting more than 10% of people [1]. It affects people physically [2], mentally [3], and socially [4], leading to “poorer health outcomes, increased hospitalisation, and lower quality of life” [5]; anxiety whose prevalence among patients with CKD was 19%, and anxiety symptoms whose occurrence was 43% [6]. Depression, a common mental disorder, according to the World Health Organization, “is characterised by sadness, loss of interest or pleasure, feelings of guilt or low self-worth, disturbed sleep or appetite, feelings of tiredness, and poor concentration” [7]. Approximately 280 million people have depression worldwide [8]. The global depression levels are an estimated 3.8% of the population, including 5.0% of adults and 5.7% of adults aged > 60 years [8]. The overall depression levels in Trinidad and Tobago are unavailable. However, the levels vary from 25.3% ± 2.37% among adolescents [9] to 40% among stable cardiac clinic attendees [10]. Depression among patients with kidney diseases accounts for 20–25% of the adult population globally [11]. The prevalence of clinical depression (Patient Health Questionnaire, PHQ > 9) among patients with CKD is 39% [12] and 46% [13] in the USA and the UK, respectively. Research by Agarwal et al. revealed the prevalence of depression among haemodialysis patients to be 78%, 65%, and 51% using PHQ-9, HAD-17, and ICD-10, respectively. [14].

Risk factors for depression include “female sex, financial difficulties/low socioeconomic status, stressful life events, lack of social support, serious or chronic illness, and a history of eating disorders” [15], many of which are common in our population.

Predictors of depression vary with socio-cultural and economic contexts. Tannor et al. identified predictors of CKD with diabetes mellitus and hypertension as increasing age, low educational status, increased duration of hypertension, and use of herbal preparations [16]. According to Yang Meng et al., independent predictors of depression in haemodialysis patients were lower monthly family income, more comorbidities, and a higher degree of pruritus. [17].

This study aimed to determine depression levels among patients with CKD and its associations and predictors.

## Methods

This cross-sectional study comprised patients with kidney diseases attending CKD clinics at a public health institute. The institute is a 325-bed hospital providing a multitude of services, including both peritoneal and haemodialysis, emergency, and follow-up care in two kidney clinics, each of which attends to approximately 40 to 50 patients per clinic day [18] conducted once per week. The patients are referred to the clinic primarily because of elevated serum creatinine (a marker of CKD), apart from leg swelling and proteinuria. CKD was defined as a decrease in the renal creatinine filtration rate (estimated glomerular filtration rate - eGFR < 60 mL/min/1.73 m^2^) [19] lasting more than 90 days. [20] eGFR is used to determine the presence of CKD (CKD stages 3–5) [20]. eGFR [21] can be classified into 5 stages: Stage 1 CKD: mild kidney damage, estimated GFR (eGFR) ≥ 90 mL/1.73 m^2^; Stage 2 CKD: mild loss of kidney function, eGFR ranging from 60 mL/1.73m^2^ to 89 mL/1.73m^2^; Stage 3a and 3b CKD: mild to severe loss of kidney function, eGFR ranging from 30 mL/1.73m^2^ to 59 mL/1.73 m^2^; Stage 4 CKD: severe loss of kidney function, eGFR ranging from 15 mL/ 1.73m^2^ to 29 mL/1.73m^2^; and Stage 5 CKD: Kidney failure or end-stage renal failure, eGFR < 15 mL/ 1.73 m^2^ [22].

Participants were selected by convenience sampling because of the challenges of obtaining a random sample. Furthermore, a sample size could not be determined primarily because of the absence of a formula to compute sample size from a non-random sample. The questionnaire was piloted among kidney and non-kidney patients and edited based on feedback from interviewees and the Ethics Committee. Submission of the results was delayed beyond the intended date due mainly to unforeseen administrative issues. There were no missing data except for one participant whose PHQ-9 score could not be computed because of missing information. The inclusion criteria were as follows: age > 18 years and being enrolled in the clinic for at least 12 months. Patients who were uremic, confused, or unable to communicate coherently were excluded from the study. Patients who visited the kidney clinic were briefed on the nature of the study and invited to participate. They were assured of the confidentiality, anonymity, and privacy of data. All participants gave their informed consent to participate in the study .

Data were collected from 01 August 2017 to 30 September 2017 using a pre-tested questionnaire. The data collection instrument was a 24-item questionnaire consisting of sociodemographic variables (12), medical diagnosis/history (2), cardiovascular history (1), and the nine-item PHQ-9 for depressive symptoms. Depression is ideally best diagnosed using the Diagnostic and Statistical Manual of Mental Disorders, Fifth Edition (DSM-5) [23]. However, PHQ-9 was used because of its brevity and simplicity, and according to Kroenke et al., it is also “a reliable and valid measure of depression severity” [24]. PHQ-9 (comprising nine questions) has a reported sensitivity of 54% and specificity of 90% for scores ≥ 10 [25].

**Fig. 1:**
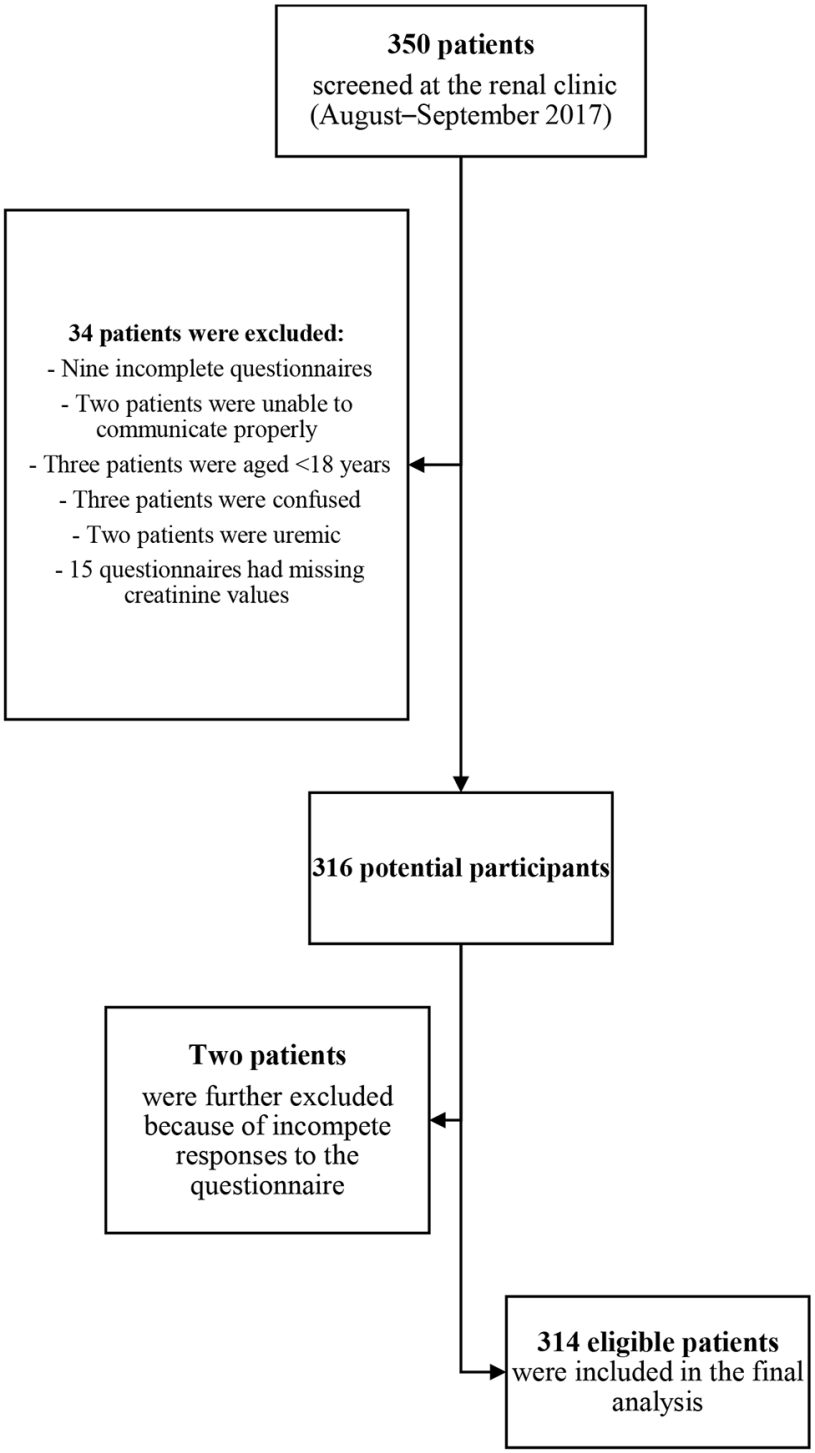
Flow diagram for participant selection

PHQ-9 data were collected via face-to-face interviews after obtaining verbal consent, whereas other relevant data were extracted from the patient records. Overall, 350 patients were selected for participation; 36 (10.3%) were excluded, resulting in a convenience sample of 314 participants. Figure 1.

Each PHQ-9 question was scored from 0 (*not at all*) to 3 (*every day*), and the individual scores were added to obtain a total PHQ-9 score (range 0–27) for each participant, with higher scores signifying worse depression. These scores were used to classify the participants according to the level of depression using the ranges provided with the data entry codes (Table 1).

**Table 1:** Categorization of the level of depression

Score range	Depression category	Code
0–4	No depression	0
5–9	Mild (*Non-clinical*)	1
10–14	Moderate (Clinical; *non-symptomatic*)	2
15–19	Moderately severe (Clinical; *Symptomatic* [26]	3
20–27	Severe (Clinical; *Symptomatic*)	4

The following definitions were used to facilitate data collection: Obesity and Hypercholesterolaemia (self-reported), Hypertensive if previously diagnosed, and currently under antihypertensive medications, in accordance with the American College of Cardiology Guidelines [27]. Cardiovascular disease (coronary artery disease and/or stroke) was determined by a “history of coronary heart disease (angina, myocardial infarction) verified through medical records of a prior episode and confirmed by work-up including electrocardiography, echocardiography, and exercise treadmill test” [28]. Physical inactivity was defined as not performing moderate to vigorous activity for at least 150 min per week; participants classified into this group were categorised as “physically inactive” [29]. Clinical depression was defined as a total PHQ-9 score ≥ 10 for all nine questionnaire items [24]. Because of the challenges in applying the definition of smokers, i.e., someone who has smoked more than 100 cigarettes in their lifetime and has smoked in the last 28 days [30], we identified smokers as persons who self-reported smoking up to the time of presentation in the clinic.

### Database and data analysis

We used Microsoft EXCEL version 10 to create a database, which was imported into SPSS, Version 21, to develop the SPSS database that was used for descriptive and inferential analyses.

Only the researchers and research assistants could access these databases. The descriptive data analysis methods included frequency, percentage distribution tables, graphs and/or charts, sample proportions or percentages for qualitative variables, and the measures of central tendency (*minimum, median, maximum, mean and standard deviation*) for quantitative variables. The inferential methods included 95% confidence intervals (CI), hypothesis testing (Z-tests for the equality of two proportions, *t*-test for the equality of two means, analysis of variance (ANOVA), chi-square tests of association, binary, ordinal, and logistic regression to identify the predictors of depression. All hypotheses were assessed at a 5% level of significance.

Ethical approval was obtained from the Ethics Committee of the South West Regional Authority.

## Results

All 314 patients completed the full face-to-face interview; however, data needed to determine depression status (PHQ 9 -score), 7 (2.2%) patients were either incomplete; or missing altogether from their respective files. Cronbach’s alpha, the measure of the PHQ-9 questionnaire reliability, was 0.745, which is indicative of good internal consistency of the responses.

### Demographics

**Fig. 2:**
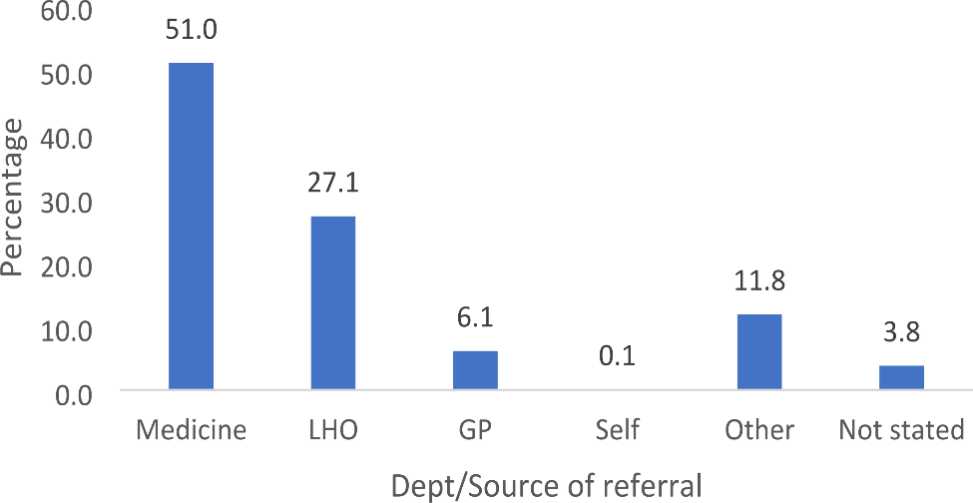
Sources of participant referral

The majority (n = 160; 51.0%) of study participants were referred by the Medical Department (Fig. 2).

They were predominantly male, over 60 years of age, of Indo-Trinbagonian ethnicity had *family support* and/or *social support* and were on *government assistance* (Table 2).

**Table 2:** Distribution of socio-demographic characteristics of participants

Variable	n	%
Gender		
Male	179	57
Female	133	42.4
Not recorded	2	0.6
Age		
< 40	13	4.1
40–50	31	9.9
51–60	51	16.2
61–70	120	38.2
> 70	91	29.0
No response	8	2.5
Ethnicity		
Afro-Trinbagonian	58	18.5
Indo-Trinbagonian	237	75.5
Mixed	16	5.1
Other	3	0.9
Family support		
No	9	2.9
Yes	297	94.6
No response	8	2.5
Social support		
No	16	5.1
Yes	288	91.7
No response	10	3.2
Financial support		
Self	64	20.4
Government assistance	175	55.7
Public assistance	9	2.9
Savings	23	7.3
Children	15	4.8
Other	16	5.1
None	3	1.0
Employment status		
Employed	39	12.4
No recent job	85	27.1
Never employed	34	10.8
Retired	121	38.5
Lost job	20	6.4
No response	15	4.8
With whom lived		
Alone	16	5.1
Spouse	152	48.4
Children	62	19.7
Spouse and children	44	14
Other	29	9.2
No response	11	3.5
Monthly income ($TT)		
< 3000	28	8.9
3000–5000	85	27.1
5000–8000	10	3.2
No response	191	60.8
Education		
Up to Primary	132	42.0
Secondary	112	35.7
Tertiary	14	4.5
No response	56	17.8

**Fig. 3:**
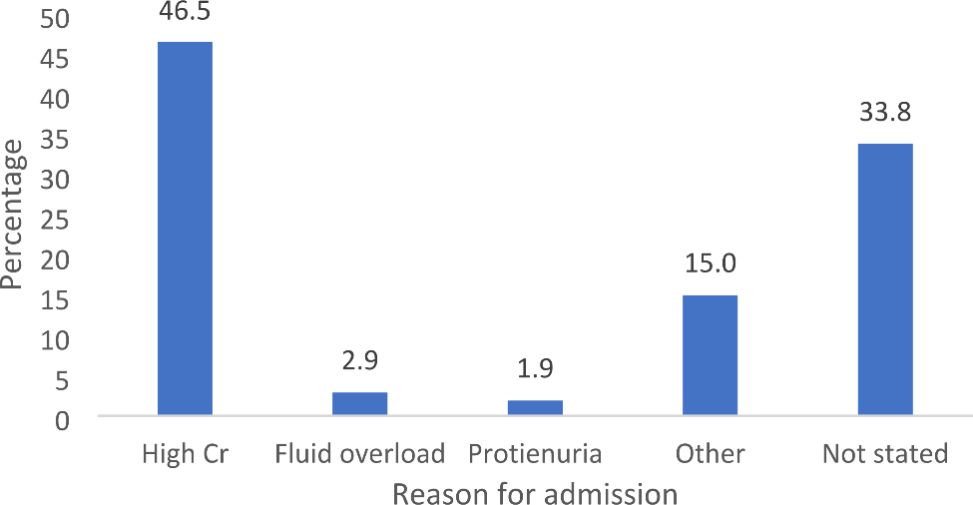
Reasons for renal clinic referral

High creatinine (n = 146; 46.5%) was the most common reason for kidney clinic referral (Fig. 3).

**Fig. 4:**
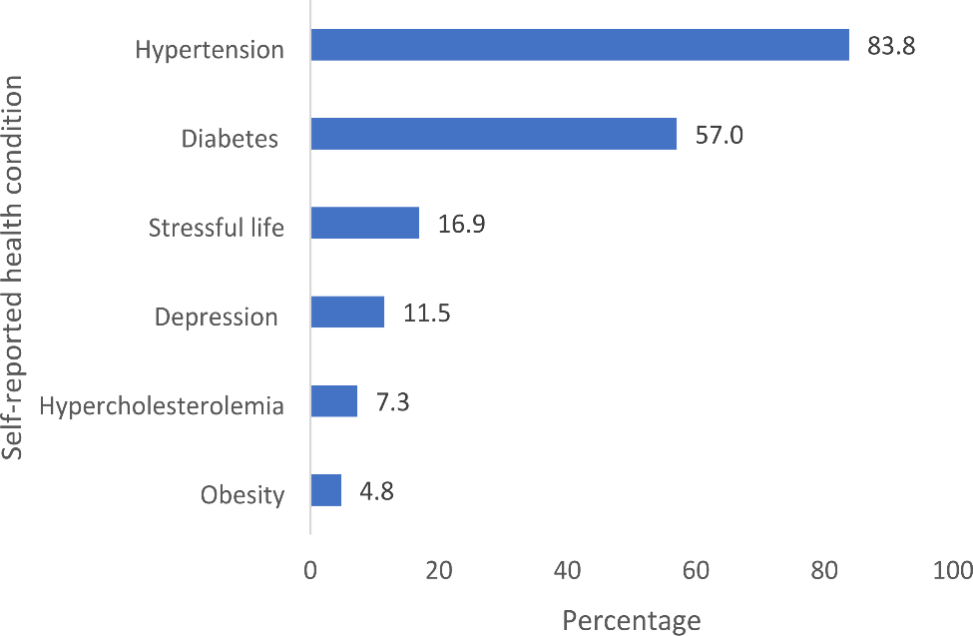
Percentage distribution of comorbidities among patients who visited the renal clinic

Hypertension was most common comorbidity (83.8%; n = 263), and obesity was the least prevalent comorbidity (4.8%; n = 15) (Fig. 4). The minimum number of conditions was 0 (n = 33; 10.5%), whereas the maximum was 5 (n = 4; 1.3%), with a mode, median, and mean of 2 (n = 138; 43.9%), 2, and 1.8 ± 1.06, respectively.

### eGFR and creatinine values

The eGFR values ranged from 4.0 mls/min/1.73 m^2^ to 130.0 mls/min/1.73 m^2^, with a median, mode, and mean value of 31, 36 (n = 11), and 33.7 (± 21.85), respectively. The mean, minimum, maximum, median, and mode values for creatinine were 3.2 (± 2.96), 0.6, 22.0, 2.1, and 1.4, respectively.

**Fig. 5:**
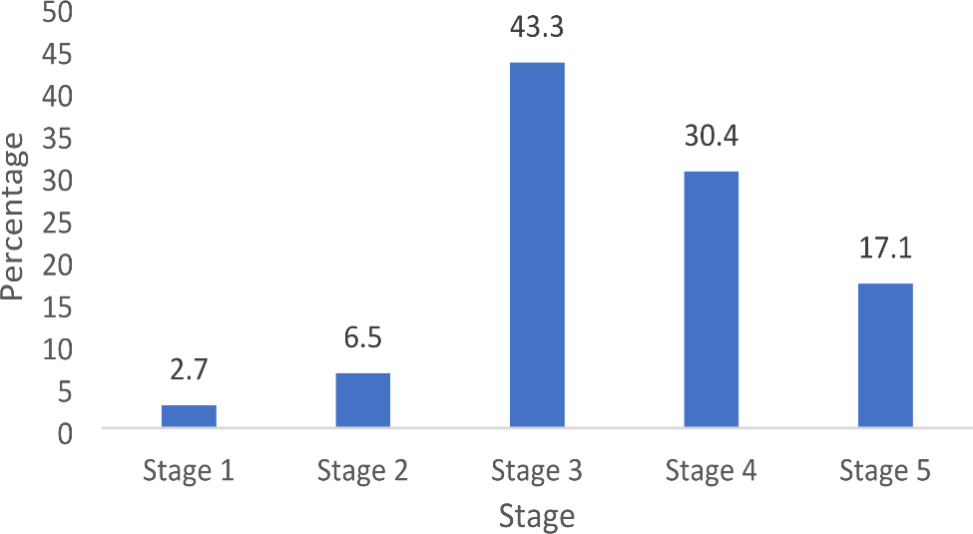
Percentage distribution of CKD stages (n = 261). CKD: chronic kidney disease

eGFR values were documented in 261 (83.1%) patients according to the CKD stage.

(Fig. 5). The prevalence of Stage 1 and Stage 5 was 2.7% (n = 7) and 17.1% (n = 45), respectively, with Stage 3 being the most prevalent (43.3%; n = 114).

### Depressive symptoms/total PHQ scores and distribution by socio-demographic variables

The mean responses for PHQ-9 questions ranged from 1.00 (*thoughts that you would be better off dead or of hurting yourself in some way*) to 1.74 (*feeling tired or having little energy*). In other words, each participant either experienced at least one of the nine symptoms or enddured them for *several days, but less than half of the days*. The overall mean of the nine items was 1.34 ± 0.249, and the minimum score was 0, similar to the median and modal scores.

### Depression scores

Total PHQ-9 scores ranged from 9 (n = 116; 37.6%) to 27 (n = 1; 0.3%). The means and standard deviations (SD) according to socio-demographic variables and the p-values obtained from using ANOVA to test for equality of within-category means are shown in Table 3.

**Table 3:** PHQ − 9 Scores (Mean and Standard deviation-SD) with p-value from ANOVA 0.3

		PHQ-9 Score		
Variable	n	Mean	SD	p-value
Gender				
Male	175	11.7	3.38	
Female	130	11.8	3.25	0.203
Age				
< 40	13	10.7	1.65	
40–50	30	11.8	3.08	
51–60	52	11.3	3.21	
61–70	115	11.4	3.48	
> 70	91	12.8	3.37	0.951
Ethnicity				
Afro-Trinbagonian	57	11.6	3.36	
Indo-Trinbagonian	232	11.9	3.37	
Mixed	16	11.1	3.03	0.567
Family support				
No	10	14.9	4.38	
Yes	296	11.7	3.26	0.014
Social support				
No	17	13.12	3.95	
Yes	287	11.7	3.30	0.140
Financial support				
None	3	13.0	6.08	
Self	64	10.7	2.93	
Government assistance	176	12.0	3.30	
Public assistance	9	15.2	2.99	
Savings	22	11.9	3.31	
Children	15	13.6	3.94	
Other	16	9.9	2.11	0.924
Diagnosed with CKD				
No	9	14.8	3.83	
Yes	283	11.8	3.33	0.316
How long since CKD (years)				
< 1 year	8	14.4	3.93	
1–5	61	13.6	3.37	
6–10	27	14.0	3.60	
>10	7	15.7	6.90	0.040
With whom lived				
Alone	17	13.6	3.41	
Spouse	151	10.5	2.29	
Children	62	12.6	3.41	
Spouse and children	44	15.1	3.91	
Other	29	10.8	2.85	0.042
Highest level of education				
Primary school	133	13.1	3.87	
Secondary school	112	11.1	2.62	
Tertiary	14	11.3	2.49	0.195
Monthly Income ($TT)				
Under 3000	28	14.1	3.37	
3000–5000	86	14.4	3.61	
> 5000	10	12.0	4.14	0.478
Employment				
Employed	39	10.25	2.13	
No recent job	83	11.1	2.57	
Never employed	35	11.4	3.13	
Retired	120	12.4	3.54	
Lost job	20	15.3	4.45	0.384
Overall	306	11.8	3.34	NA

PHQ: Patient Health Questionnaire; CKD: chronic kidney disease; and SD: standard deviation

Patients with no family support had a significantly higher mean depression score than did patients with family support (p = 0.014). However, patients who lived with spouses had a significantly lower mean PHQ-9 score than patients who lived with their children (p = 0.030) and patients who lived with their children and spouse (p = 0.002). Furthermore, patients with CKD for 10 years or more had a higher mean score than patients with CKD for less than 1 year (p = 0.042), patients with CKD for 1 to 5 years (p = 0.010), and patients with CKD for 6 to 10 years (p = 0.011).

### Depression severity: associated factors and predictors

**Fig. 6:**
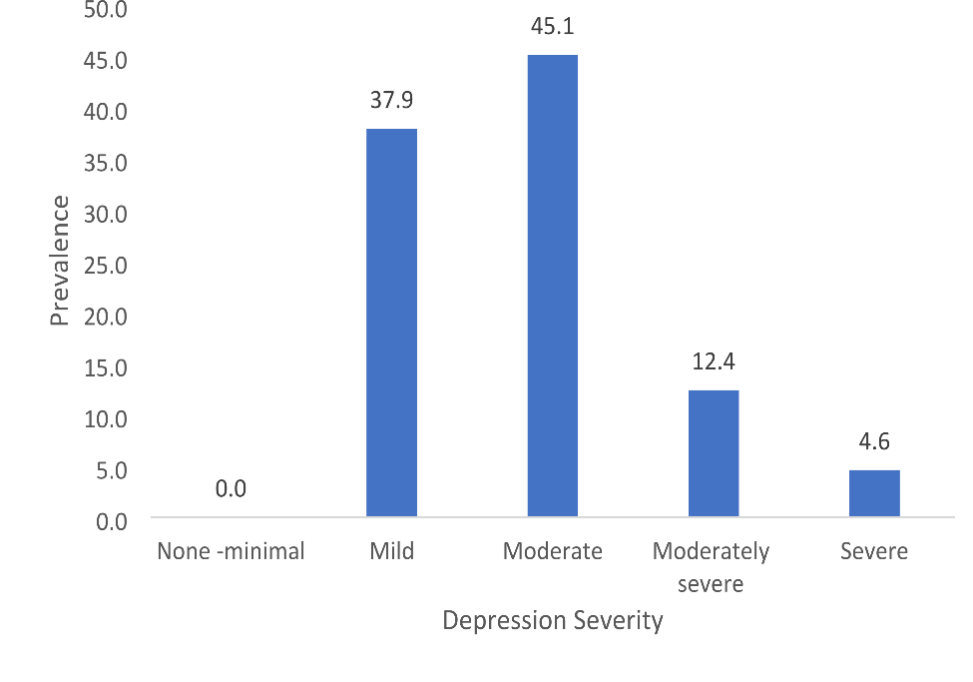
Percentage distribution of depression severity (n = 306; 97.5%)

None of the PHQ-9 scores ranged from 0 to 4, i.e., none of the patients had ***none*** to ***minimal*** depression. The majority of patients had moderate depression (non-symptomatic) (n = 138; 45.1%), and the smallest percentage had severe depression (clinical; symptomatic) (n = 14; 4.6%) depression (Fig. 6).

Pearson’s Chi-square tests of Association (Table 4) showed that, except for *gender*, each of the qualitative variables measured was associated with depression severity. Furthermore, Ordinal Logistic Regression showed that of the nine associated variables, ‘***Level of Education’***, was the only predictor of depression severity. Respective Adjusted odds ratios (AOR) with corresponding 95% CI are shown in Table 5.

Specifically, patents with Primary School education only were thirty-three times more likely to experience some level of depression compared to those with a tertiary level education. Patients with A Secondary School education were equally as likely as those with a tertiary education to experience some degree of depression.

**Table 4:** Variables associated with the level of depression severity

Variable	Chi-square	df	p-value
Gender	1.70	3	0.637
Level of education	24.29	6	≤ 0.001
Monthly income	18.36	6	0.005
Persons “Lived with”	75.29	12	≤0.001
Has family support	18.93	3	0.001
Financial support	46.74	18	≤ 0.001
Employment status	40.14	12	≤ 0.001
How long since CKD onset	89.13	15	≤ 0.001
Age group	30.77	15	0.009

CKD = Chronic Kidney Disease

**Table 5:** Predictor of depression severity:

			95% CI for AOR	
Variable	AOR	p-value	Lower	Upper
Level of Education				
Primary	33.261	≤ 0.001	6.211	178.130
Secondary	4.213	0.078	0.850	20.870
Tertiary	1			

### Depression severity according to the CKD stage

**Fig. 7:**
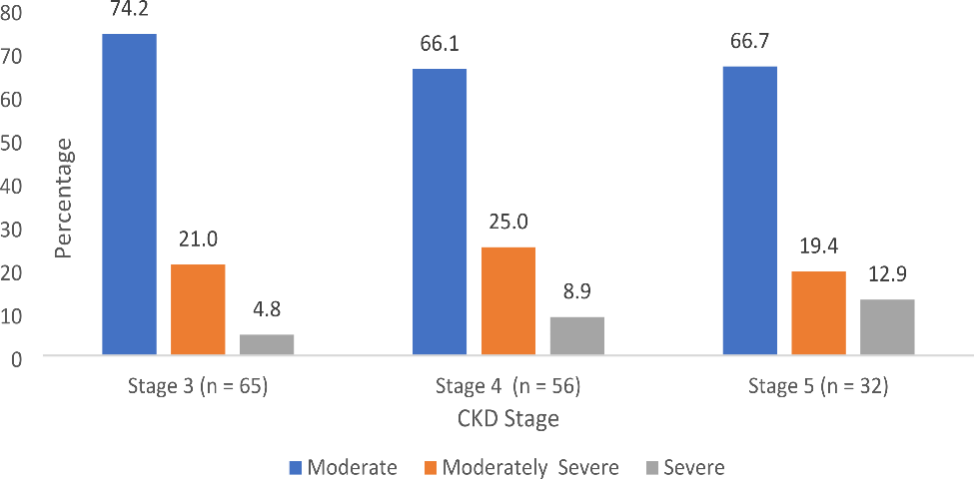
Depression severity according to the CKD stage (n = 153). CKD: chronic kidney disease

Figure 7 shows the percentage distribution of depression levels among the 153 (48.7%) patients with Stage 3 (n = 65), Stage 4 (n = 56), and Stage 5 (n = 32) CKD.

Chi-square analysis showed no association between the two variables (Chi-square: 4.248, df = 8, and p = 0.834).

Finally, the Mc Nemar-Bowker test of equality of paired proportions showed significant discordance between self-reported depression level and PHQ-9 depression classification (p ≤ 0.001). In total, 78.3% of the patients who self-reported no depression had PHQ-9 scores ≥ 10 (moderate to severe depression). Table 6 shows that of the 225 (87.2%) patients who self-reported having no depression, 202 (89.8%) were designated as having Moderate to Severe depression by PHQ-9 assessment. Table 6.

**Table 6:** Self-reported vs. PHQ-9 depression

	PHQ-9 severity designation: n (%)	
Self-reported	PHQ ≤ 9 None to Mild depression	PHQ ≥ 10 Moderate to Severe
No	23 (8.9)	202 (78.3)
Yes	1 (0.3)	32 (12.4)

## Discussion

The study population comprised principally of referrals with CKD or stages 3, 4, and 5 (90.8%), with over one-third (n = 120; 38.2%) having an age range of 61–70 years. Participants were predominantly male (57.0%), Indo-Trinidadian (75.5%), and with cardiovascular risks factors, including hypertension (83.8%) and diabetes (57%). These characteristics differ from overall population characteristics. The age-standardised global prevalence of CKD, stages 3–5 in adults aged ≥ 20 years was 4.7% in men and 5.8% in women [31]. According to the center for disease control, CKD stages 3–4 prevalence among patients with diabetes in the United States (US) was 24.5% from 2011 to 2014, and among adults with hypertension in the US adults was 35.8% from 2011 to 2014 [32].

Clinical depression.

Each participant had experienced at least one of the nine depressive symptoms or experienced them for several days but less than half of the days. More than half of the patients (62.1%) were classified as those with clinical depression: moderate (n = 138; 45.1%), moderate to severe (n = 40; 12.4%), and severe depression (n = 14; 4.6%). These findings are similar to those of a study by Gardia et al. among patients with CKD on haemodialysis which revealed an overall depression of 66% (n = 100): 28.8% had moderate depression, and 13.6% had severe depression [33]. Gardia et al. also found that depression was more prevalent in female patients (86%) as compared to male patients (57%). (*P* = 0.005).

Our study revealed that chi-square tests of association demonstrated that gender, education level, monthly income, persons “lived with”, family support, financial support, employment status, CKD duration, and age group were associated with clinical depression. These findings corroborated with other studies that revealed an association between depression and sociodemographic variables, such as income [33], employment [34], social status [35], and social support [36]. A study by Duan et al., analyzing other variables revealed “negative illness perception, low self-esteem and severe pain interference” were associated with depression among CKD patients not on dialysis. [37].

According to Pevrol et al. there is a similar associaltion between lower level of education and major depression. [38]. Patients with lower level education were significantly more likely to have major depression than those with higher education. This association of lower educational level with more depression was reported by Timmermans IAL and Widdershoven J [39]. Because depressed people with lower education may have less access to screening and treatment. [40].

Furthermore, in our study, ordinal logistic regression revealed that “***level of education”***, was the only predictor of depression severity *with greater severity associated with lower levels of education*. Other predictors of major depression in CKD, identified by Tezel and Turkistani were family support [41, 42]. Support is a major contributor to patient security [43], quality of life [44, 45], and life expectancy. The lack of patient support hinders transport support services and supplementation of treatment; thus, there is a risk of worsening depression with complications and early death [46]. Most patients in this study received financial support, followed by help from the family. These findings differ from those of Silva et al. [46] and Bapat et al. [47]. Depression can lead to poorer health outcomes [48], increased hospitalisation [49], and lower quality of life [50]. Additionally, it was discovered that medication nonadherence is linked to worsening of CKD [51].According to Meng et al., depression and worsening CKD together increase the risk of both cardiovascular and all-cause death. [52].

The difference between the prevalence of depression was insignificant among women and men (63.9% vs. 60.5%) (p = 0.637). However, Sqalli-Houssaini et al. [53] and Chiang et al. [54] demonstrated a significant association. Our findings revealed no significant association between depression severity and ethnicity (p = 0.776). Similarly, Mosleh et al. demonstrated that age was the only variable associated with depression among patients with CKD undergoing dialysis and was the only predictor of depression (OR: 1.040; 95% CI: 1.004–1.076; p = 0.027) [55]. Factors associated with depression in other populations studied include satisfaction with care [56], social support [57], and associated comorbid conditions [58]. Furthermore, researchers have reported “a higher prevalence of depression among patients with CKD without religious beliefs, no regular exercise regimen, sleep disorders, and diagnosed with stage III or higher CKD” [54].

At each stage of CKD, moderate to moderate severity of depression remained relatively the same (between 65 and 75%) except for severe depression, which seems to increase with worsening CKD – stages III to V). The chi-square analysis did not reveal an association between these variables (Chi-square: 4.248, df = 8, p = 0.834). Tsai et al., Lee et al., and Danielle et al. reported clinical depression levels of 37% [59], 47.1% [60], and 40% [61], respectively, for CKD Stages III, IV, and V. Generally, patients with CKD (Stage V) displayed the highest prevalence of clinical depression [55]; however, this finding was inconsistent. Dziubek et al. revealed a 53% prevalence of depression among patients with Stage V renal failure [62]. In this study, high levels of depression were not surprising because of the lack of quality support (financial, social, and medical), also reported by Hettiarachchi and Abeysena [63].

Further, a significant number (78.3%) of patients who self-reported no depression had at least moderate to severe depression (PHQ-9 scores ≥ 10). Self-reported depression among patients with CKD was reported to be less accurate, especially among patients with kidney failure. [64].

### Limitations

The study sample was relatively small because of resource constraints. Moreover, it was not randomised but a convenience sample. The patients had to rely on recall, which could be challenging. Apart from hypertension and diabetes, the medical history of the respondents was not appropriately documented. Further, clinical depression evaluation based on the PHQ 9 score is subjective and may underestimate or overestimate symptoms. The use of PHQ 9 is a major limitation of diagnosisng depression which requires use of the DSM-5 criterion. The PHQ 9, however has shown merits as a major screening tool. Patients with at least moderate to severe depression should have been referred for further psychiatric evaluation and treatment. Although the data is not current since a few years have lapsed since data collection, the results of this study is very relevant today since the type of patients and modus operandi of clinics remain the same.

## Conclusions

Depression and depressive symptoms are major comorbidities in patients with CKD. Furthermore, the lack of social support is a predictor of depression. There is marked discordance in the high levels of depression reported using depression tools and low levels identified from self-reporting. Self-reported evaluation can underdiagnose depression and depression severity. Scientific evaluation of depression can avoid underdiagnosis, delayed diagnosis, delayed psychiatric referrals, and definitive treatment. Routine screening for depression with adequate systems in place for management should be mandatory. Greater work is required to determine psycho-social and medical determinants of depression and quality of life among patients with CKD.

## Data Availability

The data supporting the findings of this study shall be made available from the corresponding author upon reasonable request.

## List of Abbreviations

PHQ-9: Patient Health Questionnaire-9
KDIGO: Kidney Disease Improving Global Outcomes
CKD: Chronic kidney disease
GFR: Glomerular filtration rate
eGFR: Estimated glomerular filtration rate
ESRD: End-stage renal disease
AOR: adjusted odds ratios
ANOVA: Analysis of variance
