# Correction: Depression among patients with chronic kidney disease, associated factors, and predictors: a cross-sectional study

**DOI:** 10.1186/s12888-023-05383-7

**Published:** 2023-11-27

**Authors:** Mandreker Bahall, George Legall, Carlyle Lalla

**Affiliations:** 1 University of the West Indies, Eric Williams Medical Sciences Complex, Mt. Hope, Trinidad and Tobago; 2 San Fernando General Hospital, Chancery Lane, San Fernando, Trinidad and Tobago

Correction: BMC Psychiatry 23:733 (2023)

10.1186/s12888-023-05249-y

Following publication of the original article [1], the authors identified an error in the caption of Fig. 5. The correct caption is given below:

The incorrect caption is: Percentage distribution of CKD stages (n = 261). CKD: chronic renal failure

The correct caption is: Percentage distribution of CKD stages (n = 261). CKD: chronic kidney disease.

The original article [1] has been corrected.
